# *Peganum harmala* enhanced GLP-1 and restored insulin signaling to alleviate AlCl_3_-induced Alzheimer-like pathology model

**DOI:** 10.1038/s41598-021-90545-4

**Published:** 2021-06-08

**Authors:** Rofida A. Saleh, Tarek F. Eissa, Dalaal M. Abdallah, Muhammed A. Saad, Hanan S. El-Abhar

**Affiliations:** 1 Department of Pharmacology and Toxicology, Faculty of Pharmacy, Cairo University, Cairo, Egypt; 2 Faculty of Biotechnology, October University for Modern Sciences and Arts (MSA), Giza, Egypt; 3 Department of Pharmacology and Toxicology, School of Pharmacy, Newgiza University, Cairo, Egypt; 4 Department of Pharmacology, Toxicology & Biochemistry, Faculty of Pharmaceutical Sciences and Pharmaceutical Industries, Future University in Egypt, Cairo, Egypt

**Keywords:** Cell death in the nervous system, Cognitive neuroscience, Molecular neuroscience

## Abstract

*Peganum harmala* (*P. harmala*) is a folk medicinal herb used in the Sinai Peninsula (Egypt) as a remedy for central disorders. The main constituents, harmine and harmaline, have displayed therapeutic efficacy against Alzheimer’s disease (AD); however, the *P. harmala* potential on sensitizing central insulin to combat AD remains to be clarified. An AD-like rat model was induced by aluminum chloride (AlCl_3_; 50 mg/kg/day for six consecutive weeks; i.p), whereas a methanolic standardized *P. harmala* seed extract (187.5 mg/kg; p.o) was given to AD rats starting 2 weeks post AlCl_3_ exposure. Two additional groups of rats were administered either the vehicle to serve as the normal control or the vehicle + *P. harmala* seed extract to serve as the *P. harmala* control group. *P. harmala* enhanced cognition appraised by Y-maze and Morris water maze tests and improved histopathological structures altered by AlCl_3_. Additionally, it heightened the hippocampal contents of glucagon-like peptide (GLP)-1 and insulin, but abated insulin receptor substrate-1 phosphorylation at serine 307 (*p*S307-IRS-1). Besides, *P. harmala* increased phosphorylated Akt at serine 473 (*p*S473-Akt) and glucose transporter type (GLUT)4. The extract also curtailed the hippocampal content of beta amyloid (Aβ)42, glycogen synthase (GSK)-3β and phosphorylated tau. It also enhanced Nrf2, while reduced lipid peroxides and replenished glutathione. In conclusion, combating insulin resistance by *P. harmala* is a novel machinery in attenuating the insidious progression of AD by enhancing both insulin and GLP-1 trajectories in the hippocampus favoring GLUT4 production.

## Introduction

Insulin is conveyed from the periphery through a blood–brain barrier (BBB) transporter system to different brain regions^1^. Insulin receptor (IR) is densely expressed in the hippocampus, hypothalamus, and cerebral cortex^2^, where the de novo synthesis of insulin takes place^1,3^. In the brain, insulin signaling plays a crucial role in cognition, where insulin/IR interaction activates signal transduction cascades that augment synaptic plasticity mechanisms^4^. Besides the involvement of insulin and its trajectory in memory consolidation, the glucagon-like peptide (GLP)-1, another peptide hormone, plays a crucial role in maintaining the physiology of the central nervous system^5^. This hormone can also pass BBB and interacts with its widely expressed receptor in different brain regions^6^. GLP-1 has documented its capacity to enhance learning and memory, as well as to mediate neuroprotective effects among its pleiotropic pharmacological potentials^7^. Previous studies revealed that GLP-1 prompts the insulin signaling cascade to be one of its neuroprotective mechanisms^7,8^.

Over the past decade, hippocampal insulin resistance has been considered a major incentive for Alzheimer’s disease (AD) pathogenesis^9,10^. Currently, it has widely been recognized that AD may be a brain type of diabetes mellitus (DM) or “type 3 diabetes”, combining insulin deficiency with insulin resistance^11^. An early investigation probed by Steen et al.^12^ demonstrated an irregular neuronal insulin and insulin growth factor (IGF)-I/-II signaling mechanisms in AD brains. In augmentation, the amyloid beta (Aβ) protein aberrantly interferes with the insulin signaling cascade and acts as a nexus between insulin resistance and cognitive impairment in AD^13,14^. This link, hence, highlights the mutual causality between insulin resistance and Aβ oligomerization^15^ that consequently fosters mitochondrial dysfunction to induce oxidative damage^16^ and initiates membrane-dependent Aβ oligomerization mediated lipid peroxidation^17^. Oxidative stress promotes serine phosphorylation of insulin receptor substrate-1 (IRS-1) at 307, thereby progressing insulin resistance metabolic dysfunction in the brain^18^. On the other hand, GLP-1 receptor agonists reduced AD pathologic markers and improved memory in the absence of diabetes experimentally^19^; recently, a randomized double-blind placebo-controlled trial reported that long-term treatment with dulaglutide, a long-acting GLP-1 receptor agonist, reduced the hazard of substantive cognitive impairment in type 2 diabetic patients^20^.

*Peganum harmala* (*P. harmala*) L. is a traditional plant that belongs to the family of *Zygophyllaceae* and it is popularly known as Harmal or Haramlaan in North Africa^21,22^, but it is also well-adapted to other dry zones, including the Middle East, India, Iran, and Mongolia^23,24^. In Egypt, it has been used for centuries in folk medicine predominantly in the Sinai Peninsula, where the plant seeds were consumed as an analgesic and the smoke emitted from its burning leaves was inhaled for the relief of headaches and CNS disorders^22^. Phytochemical studies have established that *P. harmala* has an abundance of β-carboline alkaloids, mainly greater in the seeds^23,25^. These include harmine, harmaline, harmalol, norharmane, and harmane^25^; allowing *P. harmala* to have a wide array of therapeutic activities^24^. Studies have confirmed that harmine and harmaline retain an acetylcholinesterase inhibitory effect and an antioxidant activity, to afford a potential in AD treatment^25,26^. Moreover, harmine and harmaline exhibited an antidiabetic action, when tested in a streptozotocin rat model, an effect that was attributed to enhancing insulin sensitivity^24,27^.

In view of the aforementioned data, this study investigated the potential modulatory effect of a standardized methanolic *P. harmala* seed extract against a rat model of AD underscoring its impact on alleviating insulin resistance with relation to Aβ, tau and oxidative stress.

## Methods

### Preparation of the extract

The seeds of *P. harmala* were collected in September 2017 from the mountain of Saint Katherine (Sinai, Egypt). The herb was identified by Ibrahim El Gamal [Nature Conservation Sector (NCS), Egyptian Environmental Affairs Agency (EEAA), Egypt] and voucher specimens of *P. harmala* were deposited without identification number at Saint Katherine Protectorate, NCS, EEAA, Egypt.

The crushed seeds were successively extracted with methanol 99.8% at room temperature for 96 h, using a Soxhlet extractor. The combined methanolic extract was filtered (Whatman No.1 filter paper, CAT# WHA1001325, Sigma-Aldrich Co, MO, USA), then concentrated to dryness in a rotary evaporator at 55^ο^C and preserved at 4^ο^ C till use.

### Chemical reagents for the HPLC analysis

Standard harmine (98%) and harmaline were purchased from Sigma-Aldrich Co, (MO, USA), CAT#286,044, CAT#51,330; respectively. All used chemicals and reagents for the standardization process were of HPLC grade, procured from Merck Millipore, (Darmstadt, Germany).

### Standardization of *P. harmala* extract by HPLC

The triplet analysis of the *P. harmala extract* was carried out using the μBondapak C18 Column (125A, 10 μm, 3.9 × 300 mm; WAT027324; Waters, Dublin, Ireland) and the Shimadzu (Kyoto, Japan) LC-10AD vp pump and SPD-10A vp UV/VIS detector. The mobile phase consisted of isopropanol: acetonitrile: water: formic acid (100:100:325:0.3) at a flow rate of 1 ml/min. Standard solutions of harmine (100µ/ml) and harmaline (100µ/ml) were prepared in the mobile phase and 10 µl of the prepared standard solutions and the *P. harmala extract* were injected and detected at the UV wavelength 330 nm.

### Animals

Adult male Wistar rats, weighing 180–250 g, were obtained from the Animal House Unit (AHU) of the Faculty of Pharmacy, Cairo University (Cairo, Egypt). The animals were kept under standard housing conditions of constant temperature (25 ± 2 °C) and humidity (60 ± 10%) with 12:12 h light/dark cycles. They were allowed standard rat chow diet and water ad libitum. This study abided by ARRIVE guideline^28^ and was evaluated and approved by the Research Ethics Committee (REC) at Faculty of Pharmacy, Cairo University [PT: 2071]. All experiments were carried out in accordance with relevant guidelines and regulations.

### Experimental design

Fifty-two rats were randomly allocated into 4 groups (n = 13 each). Rats in group I received saline (i.p) and served as the normal control group, whereas those in group II provided the AD model in which rats were injected with AlCl_3_ dissolved in saline (50 mg/kg/day; i.p) for 6 consecutive weeks^29^. In group III, normal rats received daily i.p saline for 2 weeks followed by daily doses of *P. harmala *per se (187.5 mg/kg; p.o) for 4 weeks^25^, while in group IV, AlCl_3_-exposed rats were treated with the oral doses of *P. harmala* 30 min after each neurotoxin injection for a period of 4 weeks, starting from the 15th day of AlCl_3_ injection in rats that showed declined cognition^29^.

### Behavioral studies

**Figure 1:**
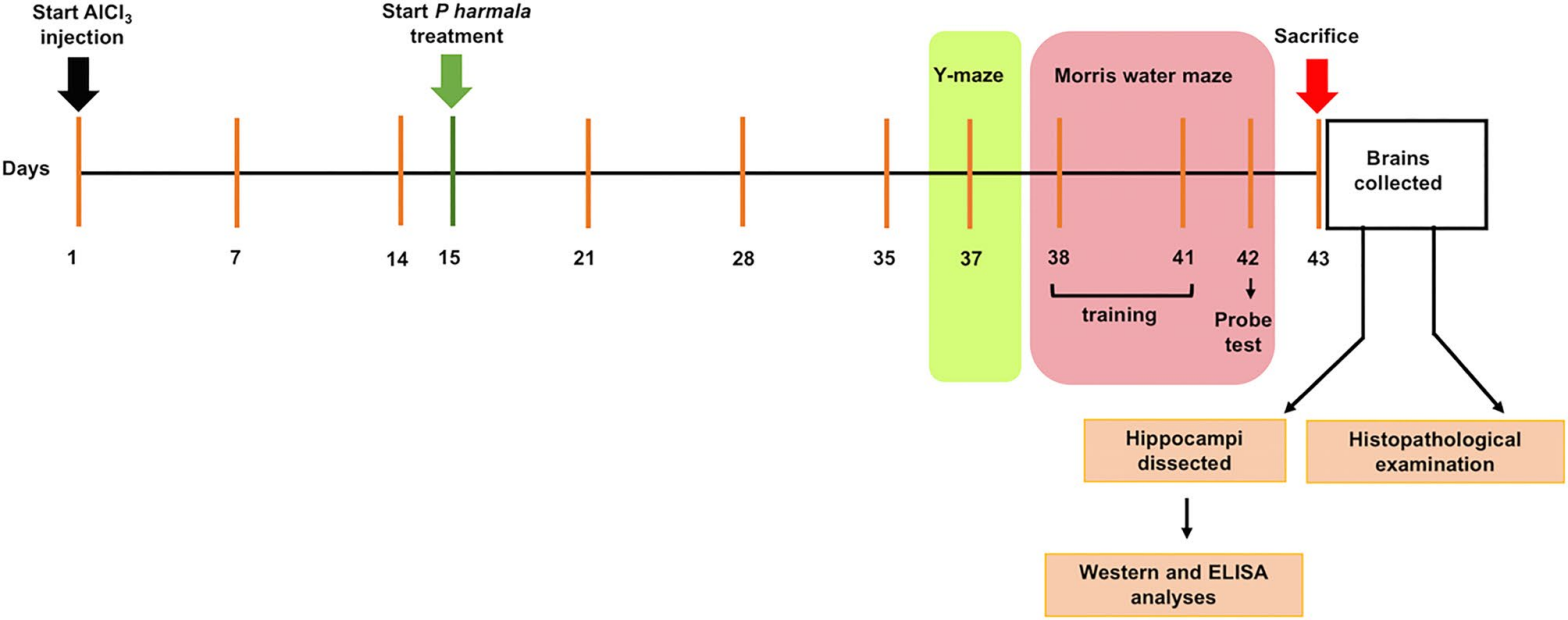
Schematic presentation of the experimental timeline. AlCl_3_ was injected on day 1 and continued till day 42 and *P. harmala* administration started on day 15. Behavioral tests were commenced with Y-maze on day 37 followed by MWM with training sessions carried on days 38–41, probe test on day 42 and animal euthanasia on day 43. *AlCl*_*3*_ aluminum chloride, *MWM* Morris Water Maze, *P. harmala* *Peganum harmala.*

Six days prior to the end of the trial, rats were subjected to behavioral tests starting with the least stressful Y-maze test on day 37, followed by Morris Water Maze (MWM) test that was carried out for 5 successive days as follows; training on days 38 to 41 and on the 42^nd^ day, the probe trial was performed (Fig. 1).

#### Y-maze task

The test is a simple and quick measure of spatial memory that depends upon the natural propensity of rats to exhibit “spontaneous alternation behavior”, in which all three arms in the Y-maze are entered successively without repeated entries^30^. The Y-maze apparatus consisted of 3 identical painted wooden arms (40 cm long, 35 cm high, 12 cm wide) designated as A, B, and C, with an angle of 120° between them. Each rat was placed in the center of the maze and was allowed to navigate freely through the maze for 5 min without cues or reinforcements, such as food or water. The number of entries/arm was recorded manually and the arm entry was considered valid when the hind paws of the animal are completely inside the arm. The total number of arm entries and the number of spontaneous alternations were counted to calculate the spontaneous alternation percentage (SAP) according to the following Eq. ^31^: *SAP (%)* = *[(number of alternations)/ (total number of arm entries − 2)]* × *100.*

#### MWM

This test is particularly sensitive to hippocampal-dependent spatial memory in rats^32^. The test was carried out following the Morris 1984 protocol^33^ using a circular pool (150 cm diameter, 60 cm high), filled to a depth of 40 cm with water (26 ± 1^ο^C). The pool was divided into 4 virtual quadrants of equal sizes and a circular transparent movable escape platform (10 cm diameter) that was submerged 1 cm below the water surface in the middle of one quadrant (i.e. the target quadrant). For the 4 subsequent days of the training session (acquisition phase), rats were exposed to 4 daily trials to find the hidden platform within 120 s. The time taken to find the platform was recorded for each trial (i.e. escape latency) and the animal was left on the platform for 10 s; however, rats that failed to allocate the platform were guided and placed on the platform for 30 s with a given latency of 120 s. On the 5^th^ day of the MWM paradigm (day 42 of experimentation), the probe test was carried out, where the platform was removed and rats were allowed to explore the maze for 120 s, in which the percent quadrant time (Q)^34^ was recorded using the following equation: *Q* = *(time spent in target quadrant (s)/120)* × *100.*

Twenty-four hours after the completion of behavioral testing, animals were euthanized using a high dose of thiopental (100 mg/kg) and brains were carefully harvested. The hippocampi were gently dissected out to be used for the determination of the biochemical parameters.

### Western blot analysis

The hippocampi from 4 rats per group were homogenized in radioimmuniprecipitation assay (RIPA) buffer (150 mM NaCl, 0.1% sodium dodecyl sulfate, 1% Triton X-100, 50 mM Tris–HCl, 1% sodium deoxycholate, pH 7.8) with protease inhibitor cocktail for Western blot analysis. The total protein content of hippocampal homogenates was assessed using the Bicinchoninic Acid Assay (BCA) assay kit (Abcam, Cambridge, UK) and then 20 µg protein aliquots of each sample were separated by SDS-PAGE then transferred into a nitrocellulose membrane using a semi-dry transfer apparatus (Bio-Rad, CA, USA). The membranes were placed in 5% bovine serum albumin in Tris buffered saline containing 0.1% Tween 20 (TBST) to block the binding sites on the membrane and to reduce background interference. After that, the membranes were incubated with primary antibodies including phospho-Akt1 (Ser473) polyclonal antibody (1:500, CAT# PA5-104,445), phospho-IRS1 (Ser307) polyclonal antibody (1:1000, CAT# 44-813G), phospho-tau (Ser262) polyclonal antibody (1:1000, CAT# 44-750G), and a β-actin monoclonal antibody as a reference protein (1:1000, CAT# MA5-15,739) overnight on a roller shaker at 4 °C. All the primary antibodies used were purchased from ThermoFisher Scientific (MA, USA). On the next day, the membranes were washed in TBST and incubated with horseradish peroxidase-conjugated secondary antibody (1:1000, CAT# ab6734, Abcam) for 1 h at room temperature. Signals were developed following enhanced chemiluminescent (ECL) reagent (CAT# 32,209, ThermoFisher Scientific). Eventually, the optical densities of the expressed protein bands were quantified via densitometry using a laser scanning densitometer (CAT# GS800, Bio-Rad). The results were normalized to β-actin and expressed as arbitrary units (AU).

### ELISA assessment

The hippocampi of 6 rats/group were homogenized in phosphate buffer and aliquoted for ELISA assay; then kept at -80^ο^C. The following hippocampal parameters were quantified using the corresponding rat-specific ELISA kits: Aβ1-42 and GLUT4 (Cusabio technology LLC, TX, USA, CAT# CSB-E10786r, CAT# CSB-E13908r; respectively); insulin, GSH, MDA and Nrf-2 (MyBioSource, CA, USA, CAT# MBS724709, CAT# MBS265966, CAT# MBS268427, CAT# MBS752046; respectively); glucagon like peptide-1 (GLP-1) and *p*S9-GSK3β (RayBiotech, CA, USA, CAT# EIAR-GLP1-1, CAT# PEL-GSK3b-S9-; respectively). The manufacturers’ instructions were precisely followed for each ELISA.

### Histopathological examination

Moreover, the whole brains of the last 3 rats/group were fixed in 10% neutral buffered formalin for 72 h followed by paraffin embedding for histopathological examination. The formalin fixed brains of each group were trimmed, dehydrated in serial grades of ethanol, cleared in xylene, and embedded in Paraplast tissue embedding media. Sagittal brain Sects. (4 µm thickness) were obtained using a rotatory microtome and were stained with Harris Hematoxylin and Eosin (H&E) staining as a general tissue structure examination. Likewise, other sections were subjected to Nissl staining using Toluidine blue to assess neuronal survival in hippocampal Cornu Ammonis 1 (CA1) and Dentate gyrus (DG) regions. Six random non-overlapping fields were analyzed blindly for counting intact neurons by using full HD microscopic camera operated by Leica application Suite for tissue sections analysis (Leica Microsystems GmbH, Wetzlar, Germany). All histopathological procedures and evaluation were performed by an external investigator in a blinded manner.

### Statistical analysis

All data sets were represented as mean ± standard deviation (SD). GraphPad Prism software 6.0 (GraphPad Software, CA, USA) was used to perform statistical analysis. Comparisons between means were carried out using one-way Analysis of Variance (ANOVA) followed by Tukey’s Multiple Comparisons test. Escape latency results from the MWM training sessions were analyzed using two-way ANOVA and followed by Tukey’s Multiple Comparisons test. Statistical significance was achieved when *p* < 0.05.

## Results

The administration of *P. harmala* to normal rats showed no substantial changes compared with their control counterparts for all measured parameters, with the exception of hippocampal GLUT4 content. Therefore, comparisons were only made with reference to the control group.

### Standardization of *P. harmala* extract

**Figure 2:**
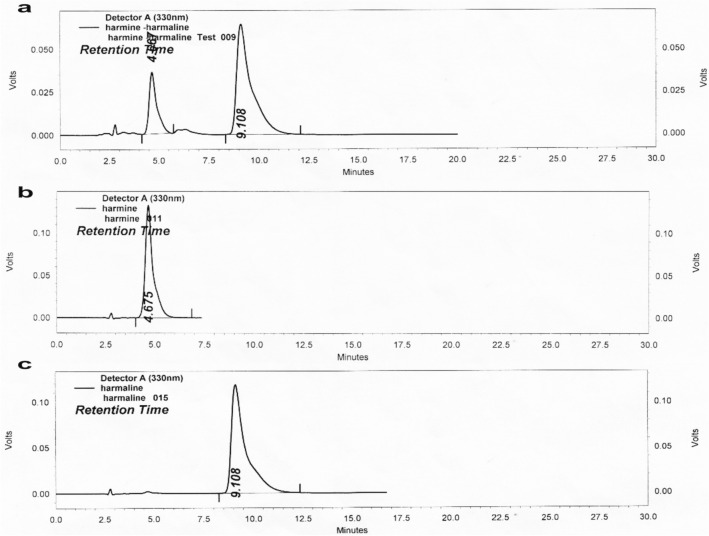
HPLC chromatogram. Graph represents (**a**) *P. harmala* extract against standard (**b**) harmine and (**c**) harmaline at 330 nm.

As illustrated in Fig. 2, the (a) harmine and harmaline in *P. harmala* extract, quantified through HPLC analysis, showed a retention time of 4.667 and 9.108 min, respectively, which was similar to those of the two standards; viz*.,* (b) harmine and (c) harmaline. The amount of harmine and harmaline present in *P. harmala* extract was estimated to be about 14 and 21% (w/w), respectively.

### *P. harmala* alleviates the AlCl_3_-induced learning and memory deficits

**Figure 3:**
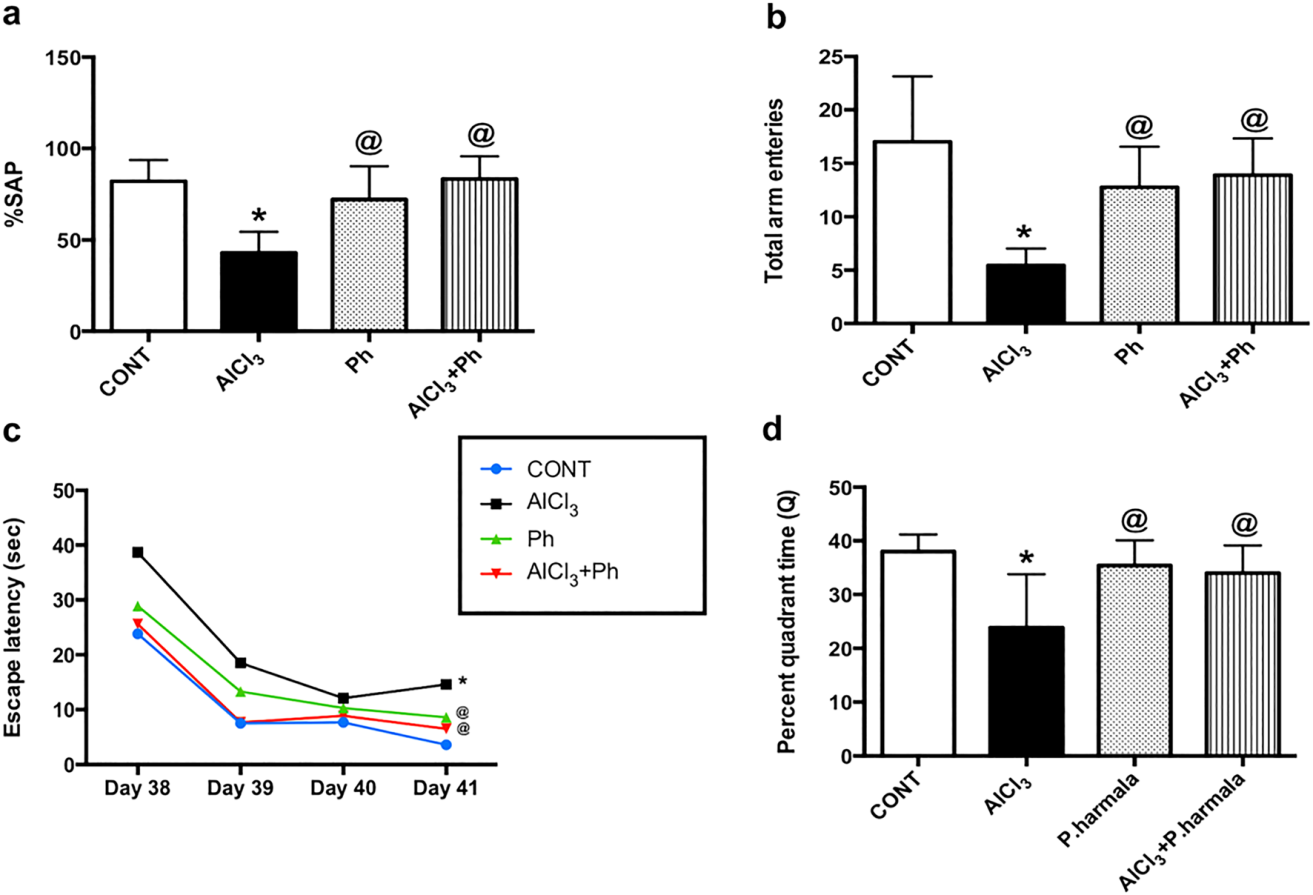
Effect of *P. harmala* on AlCl_3_-induced: (**a**) % SAP and (**b**) total arm entries in Y-maze test and (**c**) learning and (**d**) memory deficits in MWM paradigm. Values represent mean ± SD (n = 13). Statistical analysis was carried out using one-way ANOVA for %SAP and total arm entries in Y-maze and percent quadrant time in the probe MWM test, while two-way ANOVA was used for the escape latency in the training session of MWM test followed by Tukey’s Multiple Comparisons test. Significantly different from (*) CONT and (@) AlCl_3_ groups at *p* < 0.05. *AlCl*_*3*_aluminum chloride, *CONT* control, *MWM* Morris Water Maze, *Ph* *Peganum harmala*, *%SAP* spontaneous alternation percentage.

The results of the Y-maze test (Fig. 3) confirmed the spatial memory deficit and the decreased general activity in the AlCl_3_-treated rats, as evidenced by the marked inhibition in the (a) SAP and (b) total arm entries by 48 and 68%, respectively, as compared to the control group. On the contrary, post-treatment with *P. harmala* in AlCl_3_-exposed rats improved spatial memory and restored both measured parameters. Moreover, using the MWM test, the insulted rats showed a learning shortfall indicated by the prolongation of the (c) escape latency across the 4 training days in the acquisition phase compared to the control. Conversely, rats administered *P. harmala* 2 weeks after initiating AlCl_3_ injection, showed shortness in the escape latency, as compared with the AlCl_3_ group signifying improved learning. Additionally, the MWM probe test confirmed the cognitive deficit in AlCl_3_-treated rats to show compromised spatial memory that was expressed as a salient decline in (d) Q (63%) compared to the control group. However, *P. harmala* effectively ameliorated the AlCl_3_-induced memory impairment, restoring the time spent in the target quadrant to its value in normal control rats.

### *P. harmala* mitigates AlCl_3_-induced histopathological changes and intact neuron count in CA1 and DG regions of the hippocampus

**Figure 4:**
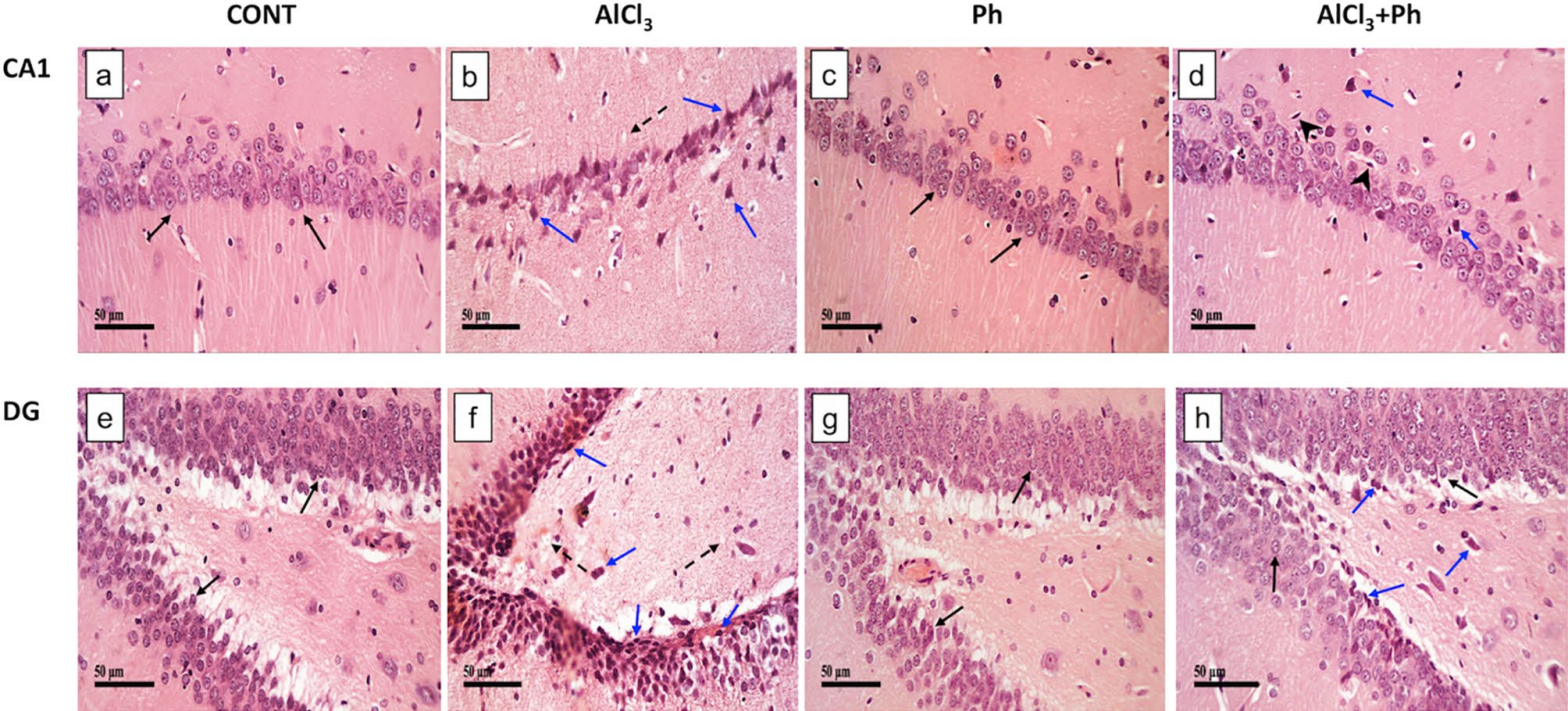
Effect of *P. harmala* on (**a**–**d**) hippocampal CA1 and (**e**–**h**) DG histopathological changes-induced by AlCl_3_ in rats. Section of (**a**) normal hippocampal CA1 area, shows intact pyramidal neurons (black arrow), while section of (**b**) AlCl_3_ group displays marked neuronal loss with varied areas of darkly pyknotic, shrunken degenerated neurons (blue arrow) with mild edema and vacuolation of neutrophils (dashed arrow). Section of (**c**) Ph group presents intact histological structures similar to normal rats and photomicrograph of (**d**) AlCl_3_ + Ph group reveals fewer degenerated shrunken pyknotic pyramidal neurons (blue arrow) combined with many intact cells and milder records of glial cell infiltrates (arrow head). In the hippocampal DG area, section of (**e**) the control group receiving saline or (**g**) *P. harmala* shows normal morphological features of hippocampal layers, including granule cells with intact nuclear details (black arrow) along with an unaltered hilar region. The section of (**f**) AlCl_3_-treated rats reveals conspicuous degenerative aberrations and many pyknotic granule cells, as well as hilar cells (blue arrow) with moderate records of edema and vacuolation of neuropil (dashed arrow). However, section of (**h**) AlCl_3_ + Ph group exhibits few scattered degenerated granule cells besides hilar cells (blue arrow) and various apparent intact granule cells (black arrow) with lower records of intracellular edema (H&E, scale bar: 50 μm). *AlCl*_*3*_ aluminum chloride, *CA1* cornu Ammonis 1, *CONT* control, *DG* dentate gyrus, *H&E* Hematoxylin and Eosin, *Ph* *Peganum harmala.*

**Figure 5:**
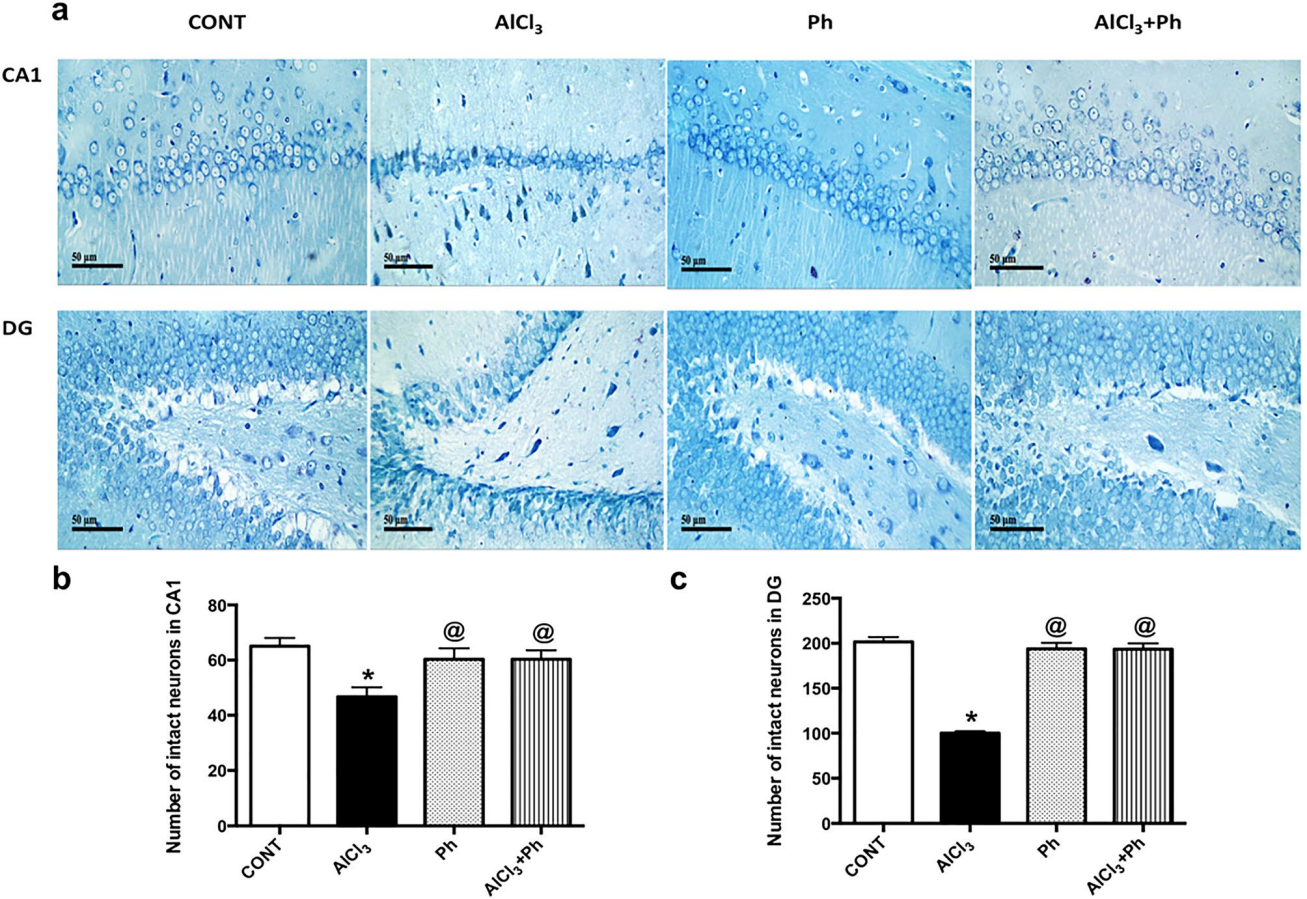
Effect of *P. harmala* on AlCl_3_-induced neuronal loss in hippocampal CA1 and DG regions. (**a**) Representative images of Nissl staining across hippocampal CA1 and DG sections (scale bar 50 μm). The lower panel shows the effect of *P. harmala* treatment on the number of surviving neurons in hippocampal (**b**) CA1 and (**c**) DG regions. Values represent mean ± SD (n = 3). Statistical analysis was carried out by one-way ANOVA followed by Tukey’s Multiple Comparisons test. (*) Significantly different from CONT and (@) AlCl_3_ groups at *p* < 0.05; *AlCl*_*3*_ aluminum chloride, *CONT* control, *CA1* cornu Ammonis 1, *DG* dentate gyrus, *Ph* *Peganum harmala.*

Compared to the normal architecture of hippocampal (a) CA1 and (e) DG areas (Fig. 4), the sections of AlCl3 rats show (b) variable alternated areas of darkly pyknotic, shrunken degenerated pyramidal neurons (CA1), and (f) granule/hilar cells (DG), besides apparent intact neurons with mild edema and vacuolation of neutrophils. However, sections of *P. harmala* post-treatment reveal fewer degenerated shrunken pyknotic cells in the (d) CA1pyramidal neurons with mild glial cells infiltrate, besides apparent intact (h) granule/hilar cells in DG with diminished records of intercellular edema. Figure 5 reveals a remarkable reduction in the number of intact neurons in hippocampal CA1 (72%) and DG (50%) regions of AlCl_3_-treated rats compared with the control. Contrarily, treatment with *P. harmala* prevented the loss of viable neurons in both regions (CA1: 1.3 folds; DG: 1.9 folds) in comparison to the AlCl_3_ group.

### *P. harmala* activates hippocampal GLP-1 and insulin signaling in AlCl_3_-induced AD-like pathology in rats

**Figure 6:**
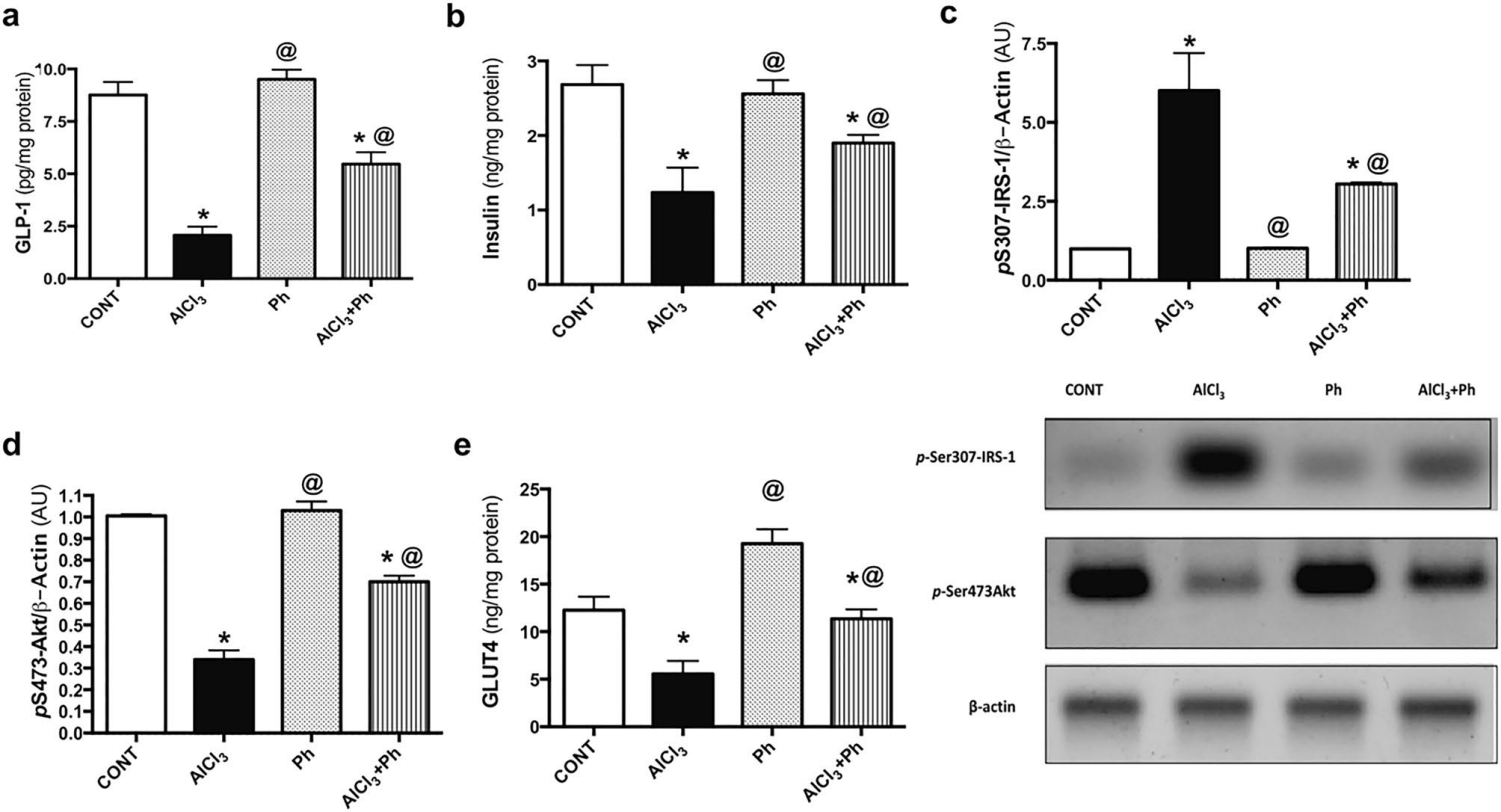
Effect of *P. harmala* on hippocampal GLP-1 and insulin signaling in AlCl_3_-induced AD-like pathology in rats. Values represent mean ± SD (n = 6/4). The blots of *p*S307-IRS-1, *p*S473-Akt and β-actin were cropped and the full-length blots are shown in the Supplementary file (Fig. S1). Statistical analysis was carried out by one-way ANOVA followed by Tukey’s Multiple Comparisons test. Significantly different from (*) CONT and (@) AlCl_3_ groups at *p* < 0.05; *pS473-Akt* phosphorylated Akt at serine 473, *AlCl*_*3*_ aluminum chloride, *AU* arbitrary units, *CONT* control, *GLP-1* glucagon-like peptide-1, *GLUT4* glucose transporter type 4, *pS307-IRS-1* phosphorylated insulin receptor substrate-1 at serine 307, *Ph* *Peganum harmala.*

As presented in Fig. 6, exposure to AlCl_3_ provoked a prominent decrease in hippocampal content of (a) GLP-1 by 74% versus control that was greatly raised by *P. harmala* (2.5 folds) compared to the insult. Additionally, AlCl_3_-treated rats halved (b) insulin content, effect that was associated with a marked elevation in (c) *p*S307-IRS-1 (6.1 folds) and the reduction in both (d) *p*S473-Akt (34%) and (e) GLUT4 (45%), as compared to the control group. In a sharp contrast, *P. harmala* extract notably boosted insulin content (1.5 folds), while reduced *p*S307-IRS-1 (51%) and enhanced *p*S473-Akt (2.1 folds), as well as GLUT4 (2 folds) when compared to the AlCl_3_ group.

### *P. harmala* ameliorates AlCl_3_-induced alterations in hippocampal Aβ42, *p*-tau, and *p*S9-GSK-3β in AD rat model

**Figure 7:**
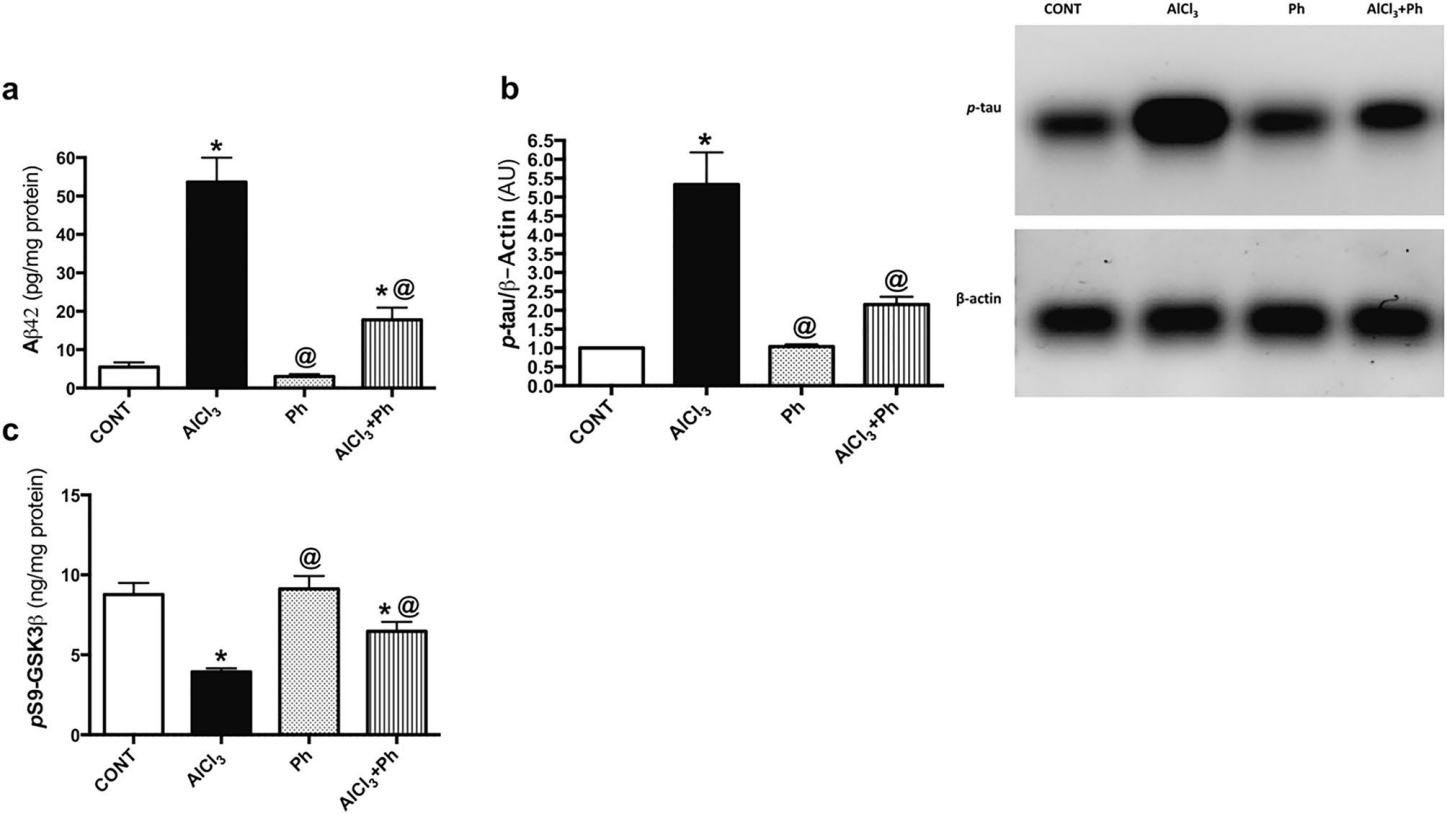
Effect of *P. harmala* on AlCl_3_-induced alterations in hippocampal (**a**) Aβ42, (**b**) *p*-tau, and (**c**) *p*S9-GSK-3β in AD rat model. Values represent mean ± SD (n = 6/4). The blots of *p*-tau and β-actin were cropped and the full-length blots are shown in the Supplementary file (Fig. S1). Statistical analysis was carried out by one-way ANOVA followed by Tukey’s Multiple Comparisons test. Significantly different from (*) CONT and (@) AlCl_3_ groups at *p* < 0.05; *AlCl*_*3*_ aluminum chloride, *CONT* control, *Aβ42* amyloid beta 42, *AU* arbitrary units, *pS9-GSK-3β* phosphorylated glycogen synthase kinase-3 beta at serine 9, *Ph* *Peganum harmala*, *p-tau* phosphorylated tau.

In Fig. 7, AlCl_3_ showed a substantial leap in the hippocampal contents of (a) Aβ42 and (b) *p*-tau to reach 9.8 and 5.3 folds, respectively, while it notably diminished (c) *p*S9-GSK-3β by 55%, as compared with the control group. However, these effects were opposed by treatment with *P. harmala,* which suppressed Aβ42 (33%) and *p*-tau (41%) and augmented *p*S9-GSK-3β (165%), as compared with AlCl_3_ rats.

### *P. harmala* abates AlCl_3_-induced oxidative stress by enhancing hippocampal Nrf2 signaling

**Figure 8:**
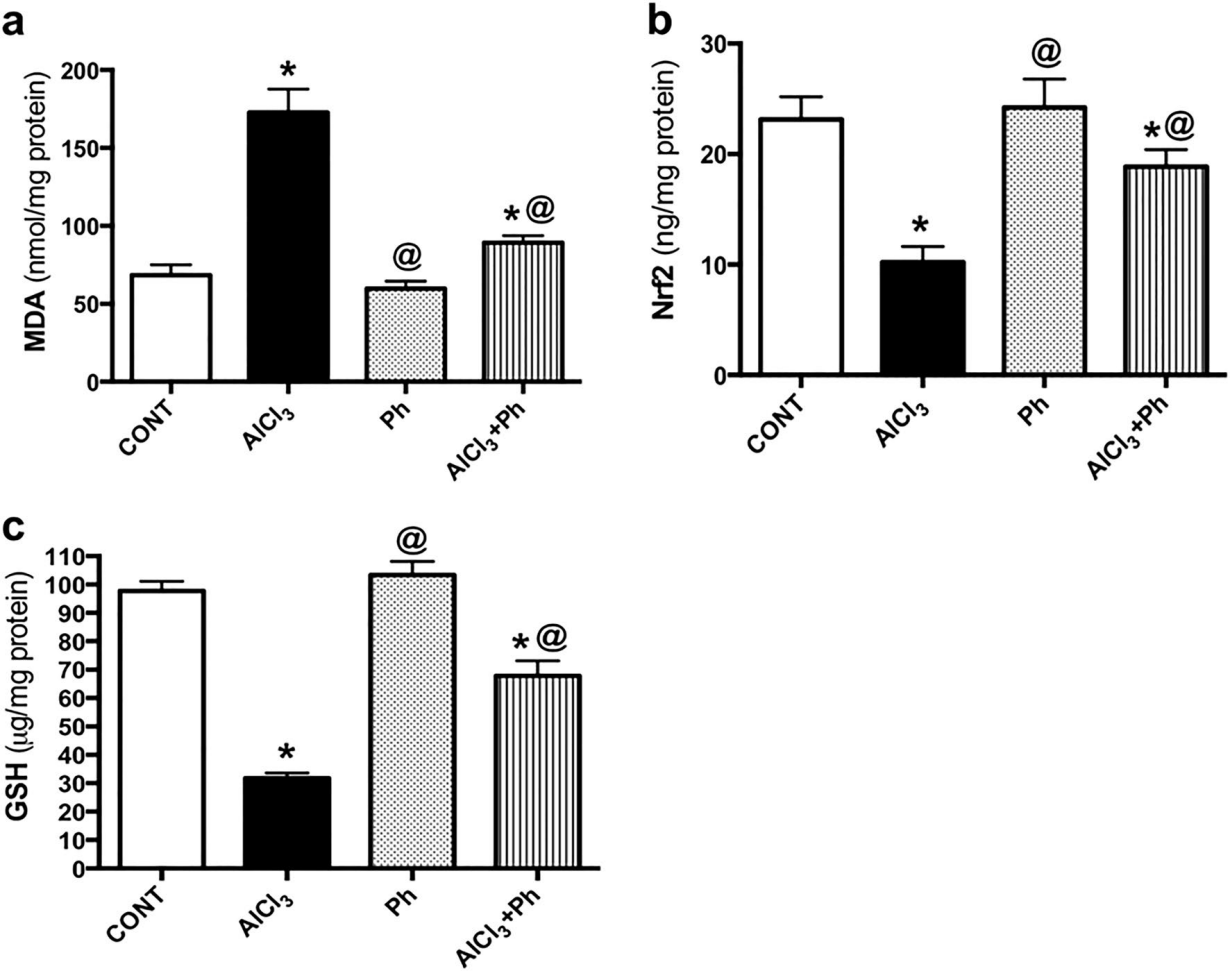
Effect of *P. harmala* on AlCl_3_-induced oxidative stress related markers (**a**) MDA, (**b**) Nrf2, and (**c**) GSH in AD rat model. Values represent mean ± SD (n = 4). Statistical analysis was carried out by one-way ANOVA followed by Tukey’s Multiple Comparisons test. Significantly different from (*) CONT and (@) AlCl_3_ groups at p < 0.05; *CONT* control, *AlCl*_*3*_ aluminum chloride, *GSH* glutathione, *MDA* malondialdehyde, *Nrf2* Nuclear factor erythroid 2-related factor 2, *Ph* *Peganum harmala.*

As shown in Fig. 8, AlCl_3_-injected rats provoked oxidative stress in the hippocampus as accentuated by the elevated content of (a) MDA (2.5 folds) in accordance with lowered contents of (b) Nrf2 (44%) and (c) GSH (32%) versus their counterpart. Conversely, *P. harmala* halved MDA, but boosted Nrf2 (1.8 folds) and GSH (2 folds), as compared with AlCl_3_-treated rats to signify its antioxidant capacity.

## Discussion

**Figure 9:**
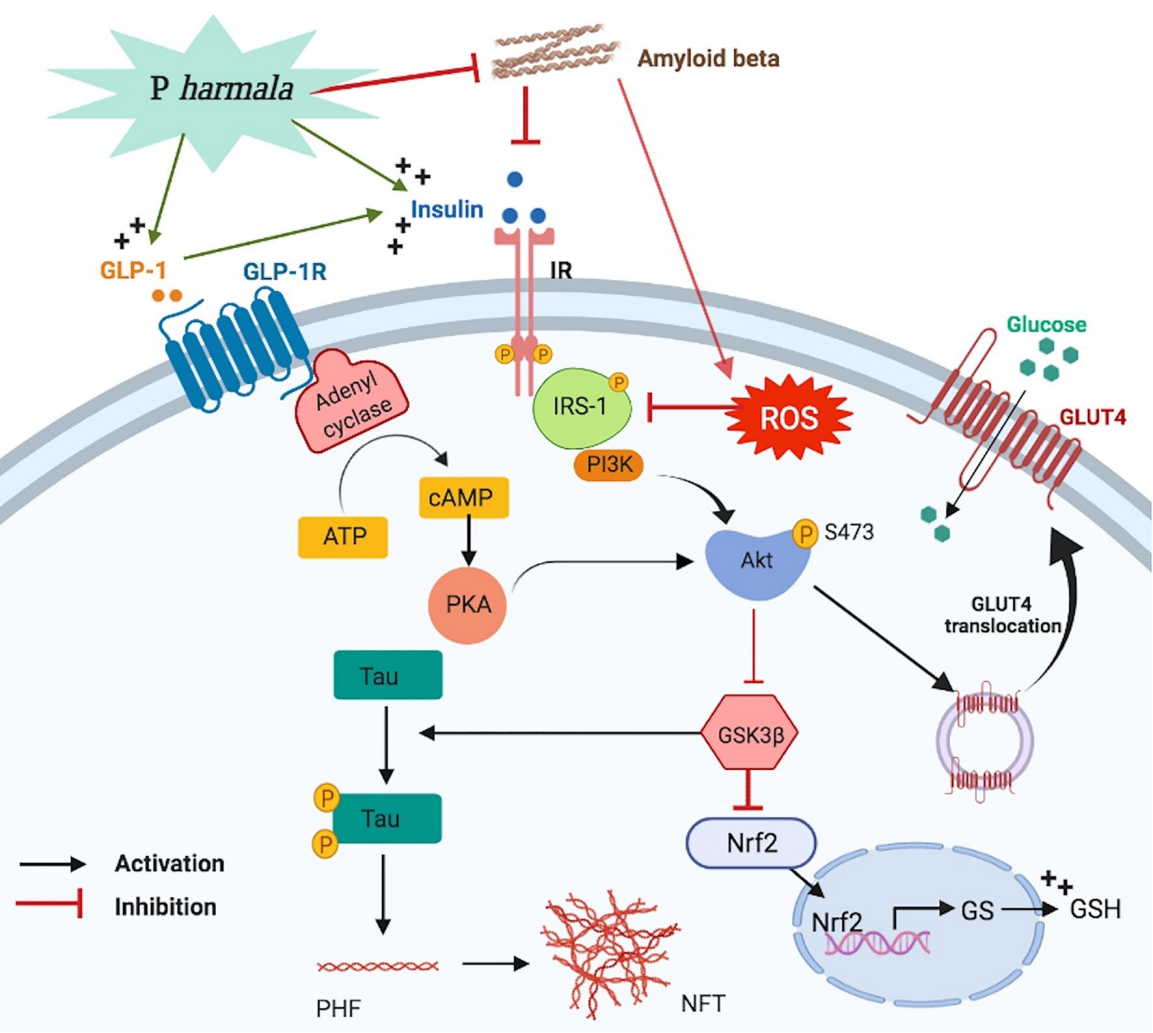
A proposed novel mechanism of *P. harmala* against insulin resistance in an AlCl_3_ sporadic model of AD. *P. harmala* restored insulin signaling via a favorable stimulation of GLP-1 and insulin/*p*-IRS-1/Akt/GLUT-4 axes versus inhibition of Aβ, GSK3β along with sequential induction of Nrf2 signaling while abating *p*-tau; hence combating AD. *Akt* protein kinase B, *ATP* adenosine triphosphate, *cAMP* cyclic adenosine monophosphate, *GLP-1/GLP-1R* glucagon-like peptide-1/receptor, *GLUT4* glucose transporter type 4, *GS* glutathione synthetase, *GSH* glutathione, *IR* insulin receptor, *IRS-1* insulin receptor substrate-1, *Nrf2* nuclear factor erythroid 2-related factor 2, *P harmala Peganum harmala*, *PI3K* phosphoinositide 3-kinase, *PKA* protein kinase A, *ROS* reactive oxygen species. “Created with **BioRender.com**".

The current report provides novel insights into the anti-amnestic effects of the methanolic extract of *P. harmala* seed in a sporadic AD model. The enhanced cognition with reduced Aβ42/tau pathology in AlCl_3_-injected rats coincided with an attenuated hippocampal insulin resistance. *P. harmala* activated the Akt trajectory to inhibit GSK3β and enhance GLUT4 via two influential molecules, namely, GLP-1 and insulin that mediated *p*S307-IRS-1 reduction (Fig. 9). The standardized extract also activated Nrf2 to enhance the brain antioxidant capacity that receded oxidative stress to additionally improve insulin sensitivity.

Intriguingly, our findings firstly validated that the insulin-signaling dysregulation in the hippocampi of rats exposed to AlCl_3_ could be ameliorated after the administration of *P. harmala* seed extract for 4 weeks. *P. harmala* commenced its central insulin sensitizing effect by enhancing the hippocampal content of insulin, results that further support a similar effect in the periphery. In a previous work, *P. harmala* seeds disclosed their hypoglycemic effect via triggering insulin secretion from pancreatic β-cells in a streptozotocin diabetic model^24^.

The enhancing effect of *P. harmala* on central insulin, recorded in our work, can be owed to the elevated hippocampal content of GLP-1, a hormone that is known to improve insulin signaling^35^. Our finding underpins the results of Gu et al.^36^, who reported that upon screening of plants for their possible dipeptidyl peptidase IV (DPP-4) enzyme inhibitory activity, harmine, a main active constituent of *P. harmala*, showed an antidiabetic DPP-4 inhibitory effect that in turn increased the endogenous levels of GLP-1^36,37^. It is worth mentioning that both GLP-1 and its receptor are decreased in AD human brain and in experimental AD models, a fact that coincide with the present findings in the current model^38^.

Following the increased hippocampal insulin and GLP-1 contents, *P. harmala* thrusted the insulin trajectory forward and extended its insulin sensitizing effect by downregulating the protein expression of *p*S307IRS-1, a leading instigator of brain insulin resistance^39^. Previously, it was reported that the decrement in IRS-1 serine phosphorylation allows the activation of the PI3K cascade^9^; this verity is confirmed herein, where the inhibition of *p*S307IRS-1 by *P. harmala* was allied by an increase in the hippocampal protein expression of the phosphorylated/activated *p*S473Akt, a downstream of the PI3K molecule. Although the central insulin sensitizing effect of *P. harmala* has not been reported before, a study conducted in 2016^27^ has shown that *P. harmala* extract mitigated palmitic acid-induced insulin resistance in muscle cells by modulating the *p*-IRS/*p*-Akt hub. Moreover, our results are compatible with an earlier study conducted by Naresh et al.^40^, which recounted that treatment of L6 skeletal muscle cells with 4-hydroxypipecolic acid, isolated from *P. harmala* seeds, stimulated GLUT4 translocation through the PI3K-dependent insulin signaling pathway.

In further support of our current results, the aptitude of *P. harmala* to increase GLP-1 adds to the activation of insulin cascade, since GLP-1 agonists, such as liraglutide and exendin-4, have activated the IRS-1/Akt trajectory in mouse models of AD^41,42^. Notably, GLP-1 mediates its effect by stimulating adenylyl cyclase to consequently trigger the PI3K/Akt signaling pathway^43^. This fact was further verified in other studies; Gupta et al.^44^ in their in vitro study revealed that treatment with exendin-4 has phosphorylated elements of the insulin-signaling hub, including Akt1. Moreover, another study showed that GLP-1 improves glucose metabolism through activating Akt^45^.

The modulation of *p*-IRS-1/*p*-Akt signaling by insulin and GLP-1 was coupled by an increase in the hippocampal content of GLUT4 to form the last step of the pathway and to highlight the beneficial role of *P. harmala* in improving cerebral insulin sensitivity that was reflected also on the refining of memory function. This notion was documented in a recent study stating that GLUT4 is one of the foremost molecules responsible for hippocampal memory and insulin sensitivity^46^. In addition to its effect on GLUT4, increased levels of GLP-1 play a critical role in improving cognition. An earlier study on GLP-1 receptor knockout mice has revealed memory impairment and cognitive dysfunction in the MWM test^47^ to coincide with the present data in AD rats. These findings, hence, emphasize the role of the active insulin cascade along with GLP-1 in the *P. harmala-*induced upturn in memory and cognition.

A crosstalk between insulin cascade and AD manifestations has been highlighted earlier, where it was identified that Aβ, a surrogate marker of AD pathology, competitively inhibits the binding of insulin to its neuronal receptors^48,49^ and interferes with tyrosine phosphorylation/activation of IRS-1^50^, while permitting its serine phosphorylation/inhibition to hinder GLUT 4 translocation^15^. Moreover, post-mortem studies^51,52^ have displayed decreased levels of insulin in different AD brain regions; besides, brain insulin deficiency was reported to impede memory, synaptic transmission, IRS-1 tyrosine phosphorylation/activation, and the activation of Akt^53^, events that contribute to AD pathology^54^. In the present work, the treatment with *P. harmala* for 4 weeks, not only activated the insulin trajectory, but it curbed also the bolstered hippocampal content of Aβ42, as well as the protein expression of the phosphorylated/activated tau, the two main molecules related to AD pathology. Hence, these effects support the folk notion about the effectiveness of *P. harmala* in easing CNS disorders and offer another mechanism to alleviate AD-like pathology besides augmenting acetylcholine^25,26^. The aptitude of *P. harmala* to turn on the insulin cascade can explain in part the decreased levels of Aβ42 and *p*-tau, where increased insulin was reported to foster Aβ clearance and prevent Aβ plaque formation^15,55^. Furthermore, the elevated insulin level triggers the tau phosphatase to deter tau pathology^56^.

Not only the increased insulin, but also GLP-1 plays a central role in lowering both Aβ42 and *p*-tau as mentioned before^57,58^. These authors displayed that different GLP-1 receptor agonists have inhibited Aβ, tau hyperphosphorylation and neuronal damage. Besides, using DPP-4 inhibitors^59,60^ or the GLP-1 analogue Liraglutide in AD animal models succeeded to attenuate the amyloid load and improve cognitive abilities and memory^61^**.**

Additionally, the *P. harmala-*induced *p*S473-Akt plays a role too, since earlier investigations confirmed that *p*S473-Akt bestows neuronal protection against AD^62,63^. A fundamental effect of the *p*S473-Akt is to phosphorylate one of its arsenal downstream molecules glycogen synthase kinase (GSK)-3β at its serine 9 residue^64^, a fact that was recorded here in the *P. harmala* treated group. The aberrant insulin signaling augments Aβ neuropathology via activating GSK3β^65^, which interferes with the two main steps responsible for amyloidogenesis^65,66^ and is considered the main kinase responsible for tau phosphorylation, a step that precedes the emergence of neurofibrillary tangles (NFT)^67^. Mutually, Aβ upregulates GSK3β activity by inhibiting its phosphorylation as evidenced from an earlier in vitro study^67^ and here. Hence, the inhibition of the hippocampal GSK-3β adds to the mechanism of the current extract in dwindling the AD pathomolecules. The activation of Akt/ GSK-3β pathway is partly attributed to the increased GLP-1, where its analogs have restored this trajectory and attenuated memory deficits in amyloid precursor peptide/presenilin 1 mice^58,68^**.**

Although harmine has been proven to effectively enhance spatial learning and memory, as shown here and hitherto^69^, Aβ was not lowered by harmine, but rather by quinazoline derivatives^70^. On the contrary, harmine is known as a potent inhibitor of dual-specificity tyrosine phosphorylation-regulated kinase 1A (DYRK1A), which plays a central role in tau hyperphosphorylation^71,72^ to explain partially the current inhibition of *p*-tau by *P. harmala.*

From another prospective, Aβ is tied to insulin resistance via inciting oxidative stress that serves as a midpoint mechanism in this trajectory. In this context, high binding protein metals tend to interact with Aβ aggregates^73^ to produce reactive oxygen species (ROS)^74^, where hydrogen peroxide (H_2_O_2_) promotes IRS-1 phosphorylation at serine 307^18^, hence adversely afflicting insulin signaling and aggravating insulin resistance. Meanwhile, as depicted from the well-recognized oxidative stress theory of AD, the neuronal lipid membrane is penetrated by Aβ oligomers to initiate and propagate lipid peroxidation by acting as sulfur free radicals, evoking thus neurotoxicity and synapse deterioration^17^. This fact is considered as an important factor in initiating neurodegeneration in both AD patients^75^ and AlCl_3_ exposed animals^76^ to validate the present Aβ-mediated cognitive decline associated by insulin resistance and oxidative stress. The latter was documented by the increased lipid peroxidation with the depletion of the defense molecule GSH and the suppression of the antioxidant transcription factor Nrf2. In our study, *P. harmala* displayed an antioxidant capacity through bolstering GSH and Nrf2 to attenuate MDA formation. Such results are supported by Li et al. (2018) who stated that harmine and harmaline reversed scopolamine-induced oxidative damage in C57BL/6 mice^77^. Of note, activating Nrf2 has been previously reported to abrogate Aβ production through blunting β-secretase expression^78^ and control GSH synthesis being an upstream regulator of glutathione synthetase^79^. Since active GSK3β causes the proteasomal degradation of Nrf2^80^, hence, *P. harmala*-mediated Akt activation/phosphorylation could be one culprit for GSK3β deactivation to enhance Nrf2, improve cognition, and reduce insulin resistance.

To this end, the therapeutic benefit of *P. harmala* extract against AD-like pathology resides in its central ability to ameliorate hippocampal insulin resistance and oxidative stress through the crossed interaction of insulin/PI3K/Akt/GLUT4, GLP-1/Akt/GLUT4 and Akt/*p*GSk3β/Nrf2 cues. The activated pathways acted on tackling Aβ aggregation, tau phosphorylation mainly by inactivating/phosphorylating GSK3β. Accordingly, the novel findings of this study propose a compelling therapeutic mechanism for *P. harmala*, beyond its traditional use, in the treatment of AD, which needs further clinical proof.

## Supplementary Information

Supplementary Information.

## Data Availability

All data generated or analyzed during this study are included in this article and its Supplementary information files.

## Abbreviations

Aβ42: Amyloid beta 42
AD: Alzheimer’s disease
Akt: Protein kinase B
AlCl_3_: Aluminum chloride
ANOVA: Analysis of variance
BBB: Blood–brain barrier
CA1: Cornu ammonis 1
DG: Dentate gyrus
DPP-4: Dipeptidyl peptidase IV
DYRK1A: Dual-specificity tyrosine phosphorylation-regulated kinase 1A
ELISA: Enzyme-linked immunosorbent assay
GLP-1: Glucagon-like peptide-1
GLUT4: Glucose transporter type 4
GSH: Glutathione
GSK-3β: Glycogen synthase kinase 3β
H&E: Hematoxylin and eosin
HPLC: High-performance liquid chromatography
IGF: Insulin growth factor
IR: Insulin receptor
IRS-1: Insulin receptor substrate-1
MDA: Malondialdehyde
MWM: Morris water maze
NFT: Neurofibrillary tangles
Nrf2: Nuclear factor erythroid 2-related factor 2
*P. harmala*: *Peganum harmala*
*p*-tau: Phosphorylated tau
PI3K: Phosphoinositide 3-kinase
*p*S307-IRS-1: Insulin receptor substrate-1 phosphorylation at serine 307
ROS: Reactive oxygen species
SAP: Spontaneous alternation percentage
SD: Standard deviation
