# Supplementary material for: Peganum harmala enhanced GLP-1 and restored insulin signaling to alleviate AlCl3-induced Alzheimer-like pathology model
Source: Sci Rep. 2021 Jun 8;11:12040. doi: 10.1038/s41598-021-90545-4 (PMC8187628; doi:10.1038/s41598-021-90545-4)

Peganum Harmala enhanced GLP-1 and restored insulin signaling to alleviate $AlCl_3$-induced Alzheimer-Like pathology model

Authors

Rofida A Saleh [1], Tarek F. Eissa [2], Dalaal M. Abdallah [1, *], Muhammed A. Saad [1,3], Hanan S. El-Abhar [1,4]

Affiliation

[1] Department of Pharmacology and Toxicology, Faculty of Pharmacy, Cairo University, Cairo, Egypt

[2] Faculty of Biotechnology, October University for Modern Sciences and Arts (MSA), Giza, Egypt

[3] Department of Pharmacology and Toxicology, School of Pharmacy, Newgiza University, Cairo, Egypt

[4] Department of Pharmacology, Toxicology & Biochemistry, Faculty of Pharmaceutical Sciences and Pharmaceutical Industries, Future University in Egypt, Cairo, Egypt.

*** Corresponding author: dalaal.abdallah@pharma.cu.edu.eg**

Supplementary figure (S1)

(A) ***p*****S307-IRS-1**

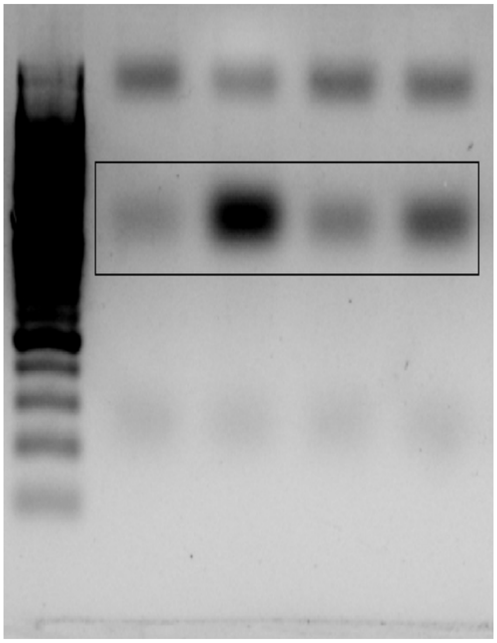

(B) ***p*****S473-Akt**

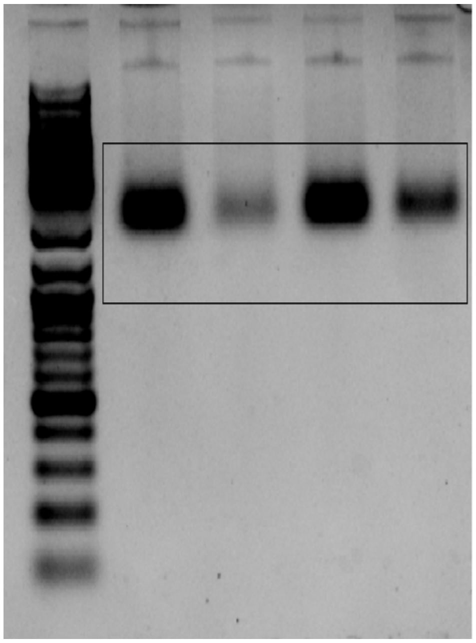

(C) β-actin

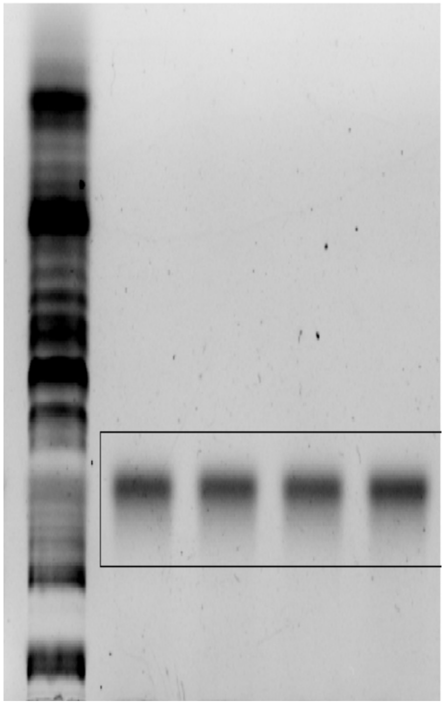

(D)*p*-tau

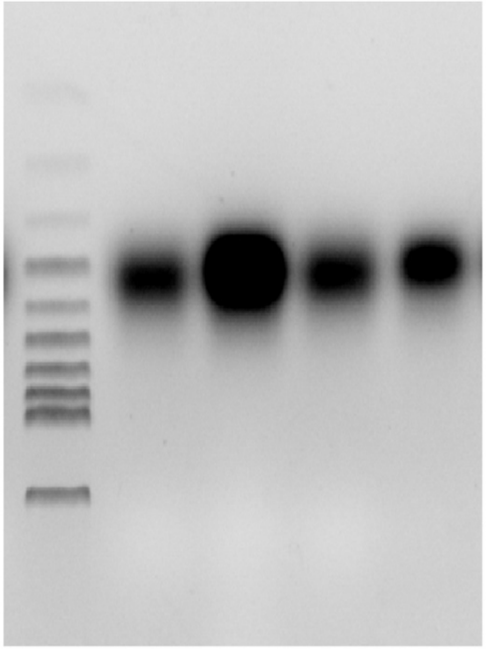

(E) β-actin

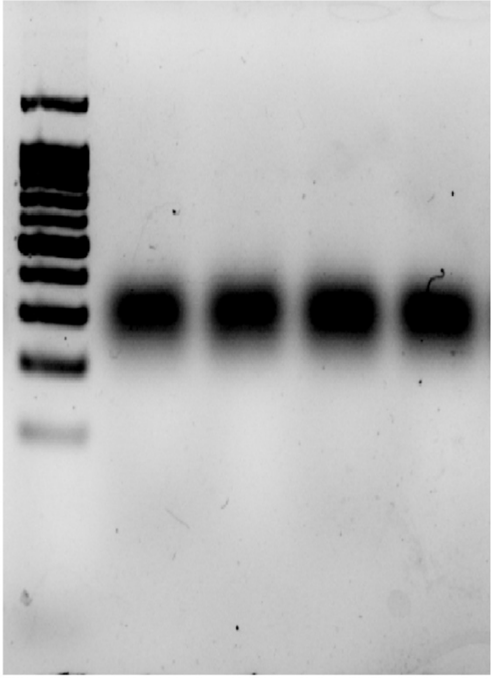

Supplement: Supplementary file 1 — Supplementary Information. [file 41598_2021_90545_MOESM1_ESM.pdf]
